# Comment on long-term ion release, fluoride recharge, pH modulation, and mechanical aging of ACP-based composite versus contemporary bioactive restorative materials

**DOI:** 10.1016/j.jobcr.2026.101464

**Published:** 2026-05-26

**Authors:** Swarupanjali Padhi, Prashant Ramdas Kokiwar, Janvi Patel, Ankita Kalra

**Affiliations:** Department of Pharmaceutics, Noida Institute of Engineering & Technology (Pharmacy Institute), Plot No.19, Knowledge Park-II, Greater Noida, India; Department of Community Medicine, Malla Reddy Institute of Medical Sciences, Malla Reddy Vishwavidyapeeth, Suraram, Hyderabad, 500055, Telangana, India; Dr. D. Y. Patil Medical College, Hospital and Research Centre, Dr. D. Y. Patil Vidyapeeth (Deemed-to-be-University), Pimpri, Pune, 411018, Maharashtra, India; Faculty of Pharmaceutical Sciences, Graphic Era Hill University, Dehradun, India; Centre for Promotion of Research, Graphic Era Deemed University, Dehradun, India

Dear Editor,

We read with interest this recent in vitro study evaluating long-term ion release, fluoride recharge, pH modulation, and mechanical aging of an experimental ACP-based composite against Activa BioACTIVE-Restorative, Cention N, and Surefil one.^1^ The integrated 12-month assessment is clinically relevant, and the conclusion that these materials are not functionally equivalent will interest restorative clinicians.

A key methodological issue concerns outcome reporting. Tables 1 and 5 are presented as cumulative ion release and cumulative fluoride re-release, yet the reported values decrease over time. Cumulative measures should not decline, so clarification is needed on whether these were interval releases after each medium refresh. This distinction is central because the inference about sustained bioactivity depends on those trajectories. Recent work in the same journal supports the remineralization potential of ACP-enriched bioactive systems, but also suggests a possible trade-off with hardness, underscoring the need for unambiguous reporting of both bioactive and mechanical endpoints.^2^

Another concern is internal consistency. Figure 2 includes microhardness although no corresponding method or result is described, Table 4 appears to duplicate the ACP row in place of another material, and the thermocycling temperatures are reported as 50 °C and 550 °C, which likely require formal correction.^1^ Clinical extrapolation also warrants caution. Recent synthesis found no significant reduction in secondary caries or retention loss with bioactive resin materials compared with conventional composites.^3^ Recent clinical studies likewise reported similar short-term outcomes for Cention N and comparable two-year posterior performance for an ionic restorative used with adhesive bonding.^4^^,^^5^

Addressing these points would improve confidence in the data, help readers separate laboratory bioactivity from clinical benefit, and better inform material selection in practice.

## Author contributions

The authors conceived the letter, critically appraised the article and literature, drafted the manuscript, and approved the final version.

## Funding

None.

## Declaration of competing interest

The authors declare that they have no known competing financial interests or personal relationships that could have appeared to influence the work reported in this paper.
